# Role of a novel immune modulating DDR2-expressing population in silica-induced pulmonary fibrosis

**DOI:** 10.1371/journal.pone.0180724

**Published:** 2017-07-10

**Authors:** Lindsay T. McDonald, Sara D. Johnson, Dayvia L. Russell, M. Rita I. Young, Amanda C. LaRue

**Affiliations:** 1 Research Services, Ralph H. Johnson VA Medical Center, Charleston, South Carolina, United States of America; 2 The Department of Pathology and Laboratory Medicine, Medical University of South Carolina, Charleston, South Carolina, United States of America; 3 The Department of Otolaryngology, Medical University of South Carolina, Charleston, South Carolina, United States of America; Federal University of Rio de Janeiro, BRAZIL

## Abstract

Micro-injuries associated with chronic inhaled particle exposures are linked with activation of the immune response and are thought to contribute to progression of fibrotic disease. In the pulmonary environment, we have previously demonstrated a heterogeneous population of circulating fibroblast precursors (CFPs), which are defined by expression of the pan-leukocyte marker CD45 and the collagen receptor, discoidin domain receptor-2 (DDR2). This population is derived from the hematopoietic stem cell, expresses collagen, and has a fibroblastic morphology *in vitro*. Herein, we demonstrate a novel subset of CFPs expressing immune markers CD11b, CD11c, and major histocompatibility complex II (MHC II). The CFP population was skewed toward this immune marker expressing subset in animals with silica-induced pulmonary fibrosis. Data indicate that this CFP subset upregulates co-stimulatory molecules and MHC II expression in response to silica-induced fibrosis *in vivo*. Functionally, this population was shown to promote T cell skewing away from a Th1 response and toward a pro-inflammatory profile. These studies represent the first direct flow cytometric and functional evaluation of the novel immune marker expressing CFP subset in an exposure-induced model of pulmonary fibrosis. Elucidating the role of this CFP subset may enhance our understanding of the complex immune balance critical to mediating exposures at the pulmonary-host interface and may be a valuable target for the treatment of exposure-induced pulmonary fibrosis.

## Introduction

Chronic exposures to inhaled particles, such as silica, are often linked with epithelial damage due to repeated physical micro-injuries to the lung epithelium. This continued injury stimulus results in initiation of pulmonary repair and activation of the inflammatory immune response [1, 2]. In pulmonary fibrosis, this repair process becomes dysregulated and develops into a persistent fibrotic response [3, 4]. While the immune cascade is known to be involved in fibrotic disease, it is unclear how the inflammatory process and epithelial damage ultimately contribute to chronicity and progression of pulmonary fibrosis. In the pulmonary microenvironment, a careful balance between immune reactivity and steady-state must be maintained due to the lung epithelial interface between the external environment and the host. While the mechanisms behind this delicate balance are not yet fully understood, immune-modulating cells involved in maintaining this homeostatic state are known to have the unique ability to respond and adapt to microenvironmental changes in response to infection, exposures, and/or self-antigens. When antigens are detected and phagocytosed by immune populations including macrophages, myeloid-derived suppressor cells, or dendritic cells, expression of major histocompatibility complex II (MHC II), and immune co-stimulatory molecules CD80/CD86 are upregulated. This process results in activation of an immune response through binding and presentation of antigen to T cells.

It was recently reported that Discoidin Domain Receptor-2 (DDR2), an extracellular matrix sensing receptor (collagen receptor) was associated with this process resulting in promotion of CD86 expression [5]. While the mechanism(s) behind this association is unclear, a cell with the ability to both sense and respond to extracellular matrix may have the potential to play a significant role in the fibrotic response to exposures. Our laboratory has previously identified a circulating fibroblast precursor (CFP) population of cells defined by the co-expression of CD45 (a pan-leukocyte marker) and DDR2 [6–8]. These cells were demonstrated to have the ability to differentiate into mature fibroblasts and promote solid tumor progression [6, 9]. In the pulmonary microenvironment, we have demonstrated a heterogeneous population of CFPs and DDR2^+^ cells that are derived from the hematopoietic stem cell, express collagen, and have a fibroblastic morphology [10]. While the ability of the CFP to give rise to fibroblasts has been established [6–8, 10], the immune contribution of this population has not yet been explored nor have these cells been examined in the context of pulmonary fibrosis. Given that the CFP has been demonstrated to contain the fibrocyte population [7] and is derived from the myeloid lineage [6], we hypothesized that the CFP may also contribute to pulmonary immune function. Therefore, in the present study, we have employed a silica exposure-induced model of pulmonary fibrosis in order to phenotypically and functionally assess the immunologic role of CFPs in disease. Herein, we have identified a subset of CFPs (CD45^+^DDR2^+^ cells) that express markers common to dendritic-like populations and other immune subsets such as monocytes and macrophages. These markers include CD11b, CD11c, MHC II, and the co-stimulatory molecules CD80 and CD86. The CFP population was skewed toward the CD11b^+^CD11c^+^ subset and demonstrated increased co-stimulatory molecule expression in silica-induced pulmonary fibrosis. In addition, this population was found to promote T cell skewing away from a Th1 phenotype toward a pro-inflammatory response in fibrotic lung, suggesting that the CFP may be involved in the inflammatory/immune balance in the fibrotic pulmonary exposure response.

## Materials and methods

### Ethics statement

Studies were conducted in strict accordance with the Veterans Affairs Institutional Animal Care and Use Committee (IACUC) under the approved ACORP #592. All efforts were made to minimize suffering. Animals were anesthetized with isoflurane prior to procedures, including euthanasia.

### Silica-induced pulmonary fibrosis

Male B6.SJL-*Ptprc*^*a*^*Pepc*^*b*^/BoyJ (C57Bl/6J) mice, aged 10–14 weeks, 28–30 g body weight, were used for all studies. These mice were purchased from Jackson Laboratories (Bar Harbor, ME, USA) and were bred in-house. Mice were anesthetized via isoflurane inhalation. A dose of 6 mg silica (Sigma-Aldrich, St. Louis, MO, USA) suspended in 60 μl of sterile 0.9% saline solution, or 60 μl of sterile 0.9% saline solution alone, was delivered via intra-tracheal instillation through a 24 gauge catheter. This dose represents ~0.2 g/Kg and dosage and delivery are based on published models of silica instillation [11, 12]. Silica suspension was vortexed immediately prior to instillation. Immediately following delivery of saline/silica the catheter was removed, animals were recovered in an upright position and were subsequently returned to their home cage. Eight weeks post-instillation, mice were anesthetized by isoflurane anesthesia and were euthanized via thoracotomy followed by exsanguination. Animals exhibiting a robust fibrotic response were selected for analysis.

### Histology

Lungs were perfused (1ml phosphate buffered saline (PBS) solution via trachea), and zinc-fixed. Paraffin sections (5μm) were stained with Picrosirius Red/Fast Green (Sigma-Aldrich) or Weigert’s Hematoxylin and either Masson’s Trichrome Stain (Richard-Allan Scientific, San Diego, CA, USA) or Herovici’s Stain (American MasterTech, Lodi, CA, USA) according to manufacturer’s protocol. Images were obtained using a Nikon TiE microscope/NIS Elements Software (Nikon Instruments, Melville, NY, USA) or a Motic Inverted Microscope (Motic, British Columbia, Canada).

### Flow cytometry/cell sorting

Lungs were digested in 1 mg/ml collagenase type I (Life Technologies, Grand Island, NY, USA, or Sigma-Aldrich) in Dulbecco’s Modified Eagle Medium, (45 minutes, 37°C). Tissues were triturated (18 gauge needle), filtered (40 μm cell strainer) and red blood cells (RBCs) were lysed (1X Pharmlyse, 10 minutes, room temperature). Cells were stained with near-IR Live/Dead Fixable Dye (Thermofisher Scientific, Waltham, MA, USA) and/or incubated with FcR block (Milltenyi Biotech, San Diego, CA, USA) (10 minutes, 4°C) before staining with primary antibodies (15 minutes, 4°C, Table 1). Analysis was performed on a BD LSR Fortessa X-20/FACS Diva 6 Software (BD Biosciences, San Jose, CA, USA). For sorting, following staining, samples were washed (PBS/DNAse I, Sigma-Aldrich). Cells were sorted using FACS Aria II Cell Sorter/FACS Diva 6 Software, (BD Biosciences). Results were analyzed using FlowJo Software V10.2 (TreeStar, Inc., Ashland, OR, USA), and gates were set based on Fluorescence Minus One controls.

**Table 1:** Antibodies used for sorting and analysis.

Antibody	Clone	Source(s)	Location	Catalog Number	Host Species	Concentration
CD45	30-F11	Biolegend	San Diego, CA, USA	103139	Rat	2 μg/ml
Discoidin Domain Receptor-2 (DDR2)	N-20	Santa-Cruz Biotechnology	Dallas, TX, USA	sc-7555	Goat	2 μg/ml
CD11b	M1/70	Biolegend	San Diego, CA, USA	101263	Rat	2 μg/ml
CD11c	N418	eBioscience	Waltham, MA, USA	56–0114	Armenian Hamster	2 μg/ml
CD80	16-10A1	BD Biosciences	San Jose, CA, USA	553768	Armenian Hamster	10 μg/ml
CD86	GL1	BD Biosciences	San Jose, CA, USA	553692	Louvain Rat	2 μg/ml
Major Histocompatibility Complex II (MHC II)	M5/114.15.2	eBioscience	Waltham, MA, USA	25–5321	Rat	2 μg/ml

### T cell co-culture

Spleens from untreated animals were homogenized and filtered (70 μm cell strainers). RBCs were lysed (ACK Lysing Buffer) (3 minutes, room temperature), and cells washed (Hank’s Buffered Saline Solution). CD4^+^ or CD8^+^ populations were isolated (magnetic bead selection kits, Miltenyi Biotech Inc). CD4^+^ or CD8^+^ T cells (1x10^5^ cells/well) were plated on 96-well plates coated with 0.1 μg/mL anti-CD3 (hamster IgG, 145-2C11, R&D Systems, Minneapolis, MN, USA) in DMEM/10% fetal bovine serum (Atlanta Biologicals, Flowery Branch, GA, USA) supplemented with 0.1 ng/ml recombinant mouse IL-2 (R&D Systems) with 1x10^4^ sorted CD45^+^DDR2^+^CD11b^+^CD11c^+^ lung cells (48 hours, 37°C, 5% CO_2_). Supernatant was collected. For activation controls, supernatant was obtained from stimulated CD4^+^ or CD8^+^ T cells (6 hrs, 2μl/ml stimulation cocktail containing phorbol 12-myristate 13-acetate (PMA) and ionomycin, eBioscience, San Diego, CA, USA).

### Cytokine analysis

Cytokine levels were quantified (mouse Th1/Th2/Th17 cytometric bead array kit, BD Biosciences) and analyzed (FACS Canto, BD Biosciences)/FCAP Array Software (Soft Flow Hungary Ltd.).

### Statistics

Comparison of CFP populations by flow cytometric analysis was based on Unpaired Student’s T-test with Welch’s Correction, where p≤0.05 was considered significant. Data represent n≥7 acquired in at least 2 experimental replicates. T cell cytokine data was compared by Unpaired Student’s T-test, where p≤0.05 was considered significant, with n≥2 for T cell isolation and n≥3 for sorted CFP populations, with at least 2 experimental replicates. Statistical analyses were performed using GraphPad Prism 5 Software (GraphPad Software, La Jolla, CA, USA).

## Results

### Silica-induced model of pulmonary fibrosis

**Fig 1:**
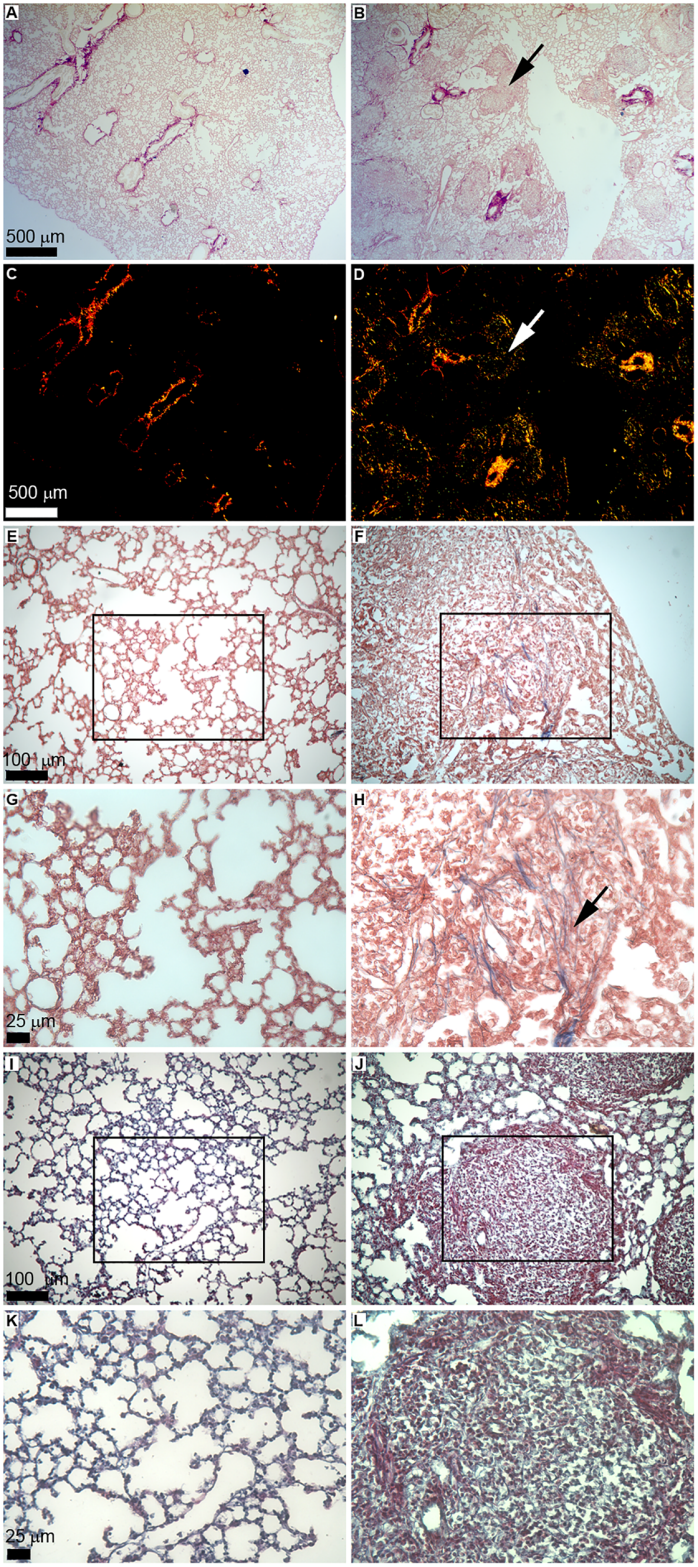
Silica-induced model of pulmonary fibrosis. Shown is representative staining of 5 μm histological sections of lung from saline- (left panels) or silica-instilled animals (right panels) at eight weeks post-instillation. Picrosirius red staining is shown in panels A-D, scale bar = 500 μm, shown in panels A and C, applies to panels A-D. (A) Representative lung architecture in saline-instilled animals. (B) Fibrotic nodules (representative nodule indicated by arrow) are evident in the lungs of silica-instilled animals. Representative Picrosirius Red staining under polarized light shows thick (red) and thin (green/yellow) collagen fibrils in lungs of saline-instilled control animal (C) and silica-instilled animal (D). Representative fibrotic nodule indicated by arrow (D). Masson’s Trichrome staining is shown in panels E-H. Lung architecture in a representative saline-instilled (E) or silica-instilled (F) animal. Scale bar = 100 μm shown in panel E, applies to panels E-F. High magnification images (boxes in (E) and (F) indicate inset) of Masson’s Trichrome stain indicate presence of collagen fibrils (representative fibril indicated by arrow) in silica-instilled animal (H) versus saline-instilled lungs (G). Scale bar = 25 μm shown in panel H, applies to panels G-H. Representative sections with Herovici’s stain from saline- (I) and silica-instilled animals (J). Scale bar = 100 μm shown in panel I, applies to panels I-J. High magnification images (boxes in (I) and (J) indicate inset) demonstrate presence of thin (blue) and thick (red) collagen fibrils in silica-instilled (L) versus saline-instilled animals (K). Scale bar = 25 μm shown in panel L, applies to panels K-L.

To address the immune role of CFPs in exposure-induced disease, a silica model of pulmonary fibrosis was employed. In this model, a silica suspension was instilled into the lungs of mice via intra-tracheal administration under isoflurane anesthesia. Eight weeks following silica-instillation, fibrotic nodules were visualized throughout the lungs and significant loss of normal lung architecture was observed versus saline only controls (Fig 1A–1L). Staining of lung sections with Picrosirius Red (PSR)/Fast Green demonstrated increased collagen deposition and presence of multiple fibrotic nodules in silica treated animals (arrow indicates representative nodule, Fig 1B) versus saline treated control animals (Fig 1A). Polarized light images (Fig 1C and 1D) indicated formation of fibrotic nodules (arrow, Fig 1D) and deposition of thick and thin collagen fibrils in silica-instilled animals (Fig 1D) versus saline controls (Fig 1C). Masson’s Trichrome stain (Fig 1E–1H) of silica exposed lungs also demonstrated increased collagen content, alveolar thickening, and multiple fibrotic nodules (Fig 1F) versus saline controls (Fig 1E). Collagen fibrils were evident in fibrotic nodules (Fig 1H) of silica-instilled animals versus saline-instilled controls (Fig 1G). Herovici’s stain (Fig 1I–1L) also demonstrated loss of normal lung architecture, apparent fibrotic nodules (Fig 1J) and increased collagen deposition in the lungs of animals with silica-induced fibrosis (Fig 1L) versus saline-instilled animals (Fig 1I and 1K).

### Contribution of a novel circulating fibroblast precursor subset in lungs

**Fig 2:**
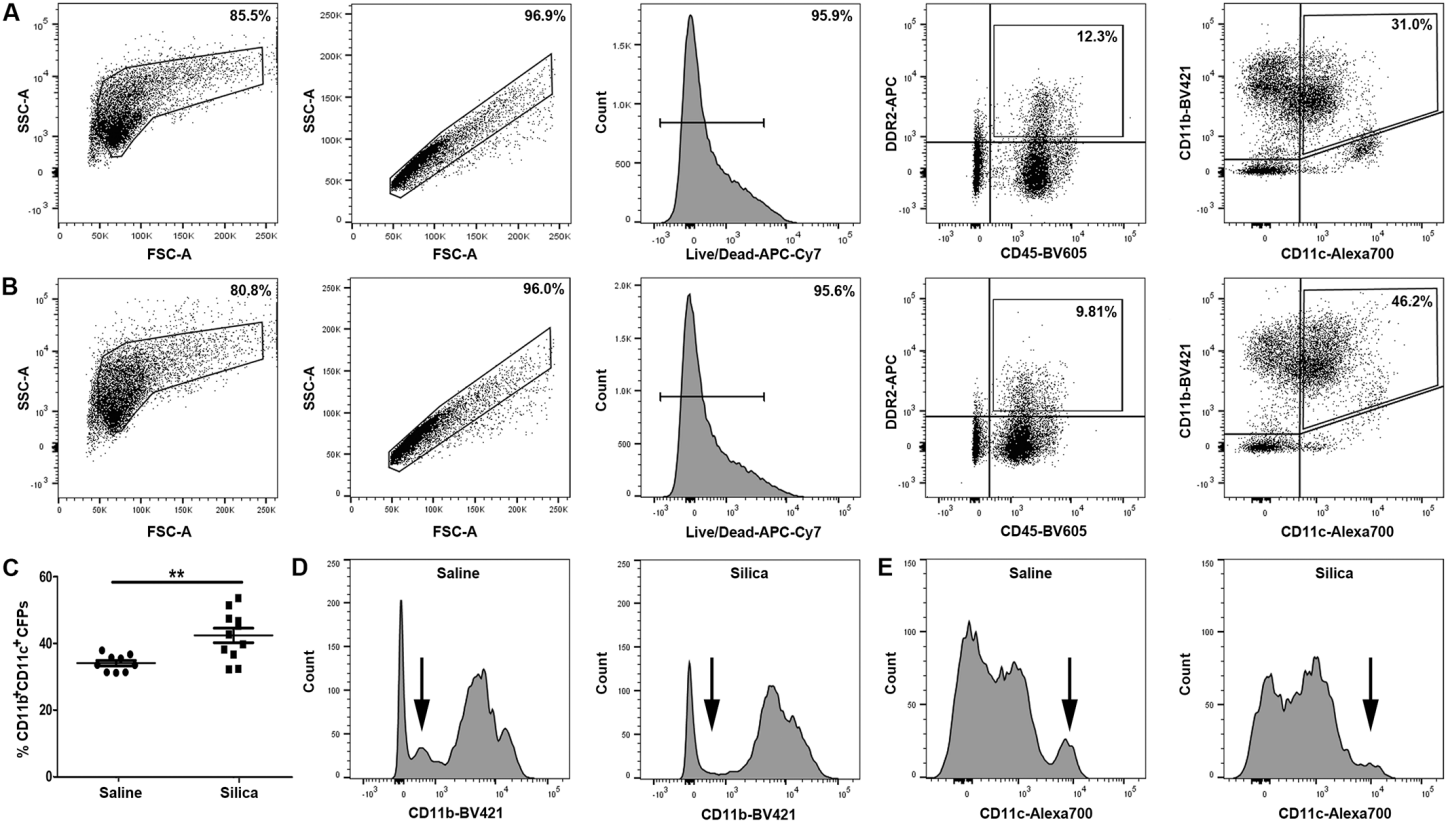
Identification of a novel circulating fibroblast precursor subset in lungs. Representative flow cytometric analysis of lung digest from (B) silica-instilled animals eight-weeks after instillation. Panels A and B depict gating strategy (left to right) of sized, single, live, CD45/Discoidin-domain receptor-2 (DDR2) cells. Expression of CD11b/CD11c on the CD45^+^/DDR2^+^ (CFP) subset is shown in the final panels of A and B. (C) Quantification of sized, single, live, CFPs expressing CD11b^+^/CD11c^+^ in saline- or silica-instilled lung digest. (D) Representative histogram of CD11b expression on sized, single, live, CD45^+^/DDR2^+^ cells from the lungs of saline-instilled (left panel) or silica-instilled (right panel) animals. Arrows indicate changing population (compare left and right panels). (E) Representative histogram of CD11c expression on sized, single, live, CD45^+^/DDR2^+^ cells from the lungs of saline-instilled (left panel) or silica-instilled (right panel) animals. Arrows indicate changing population (compare left and right panels).

In order to assess the contribution of the CFP population to fibrotic lung, lung digest from silica- or saline-instilled animals was analyzed by flow cytometry for the presence of CFPs (CD45^+^DDR2^+^ cells) (Fig 2A, saline control, and Fig 2B, silica-induced pulmonary fibrosis, fourth panels). The CFP population was reduced in the lungs of mice with silica-induced pulmonary fibrosis versus saline controls (9.076% vs. 12.48%, respectively, **p = 0.0065) (compare fourth panels Fig 2A and 2B). CD11b and CD11c expression are associated with immune populations; therefore, in order to assess the ability of the CFP population to contribute to the immune microenvironment of the lung, CFPs were examined for expression of these markers. Flow cytometric analysis of lung digest demonstrated that a subset of the CD45^+^DDR2^+^ population exhibited co-expression of CD11b^+^ and CD11c^+^ (Fig 2A and 2B, last panels). The percentage of CD11b^+^CD11c^+^ CFPs was significantly increased in the lungs of animals with silica-induced pulmonary fibrosis versus saline controls (42.41% vs. 34.11%, respectively, **p = 0.0038) (compare last panels Fig 2A and 2B, quantified in Fig 2C).

The phenotype of the CD11b^+^CD11c^+^ subset of CFPs in saline exposed lungs was predominantly CD11b^high^ (Fig 2D, left panel) and CD11c^low^ (Fig 2E, left panel). In animals with silica-induced pulmonary fibrosis, there was a loss of the CD11b^low^ population (population indicated by arrow, Fig 2D, compare left (saline) and right (silica) panels) while there was a decrease in the CD11c^high^ population (population indicated by arrow, Fig 2E, compare left (saline) and right (silica) panels).

### Phenotypic changes in immune co-stimulatory molecules on the CD11b^+^CD11c^+^ subset of CFPs in silica-induced pulmonary fibrosis

**Fig 3:**
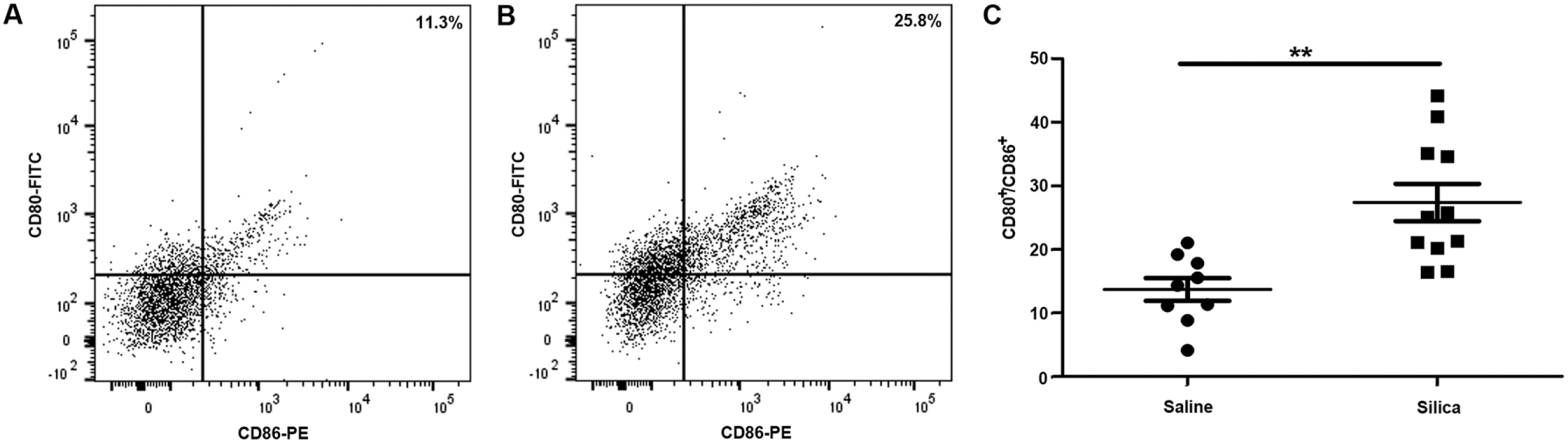
Phenotypic changes in immune co-stimulatory molecule expression on the CD11b^+^/CD11c^+^ subset of CFPs in silica-induced pulmonary fibrosis. Representative flow cytometric analysis of CD80/CD86 expression on sized, single, live, CD45^+^/DDR2^+^, CD11b^+^/CD11c^+^ cells in lung digest from saline-instilled (A) or silica-instilled (B) animals eight-weeks after instillation. (C) Quantification of CD80^+^/CD86^+^ cells gated as described above.

**Fig 4:**
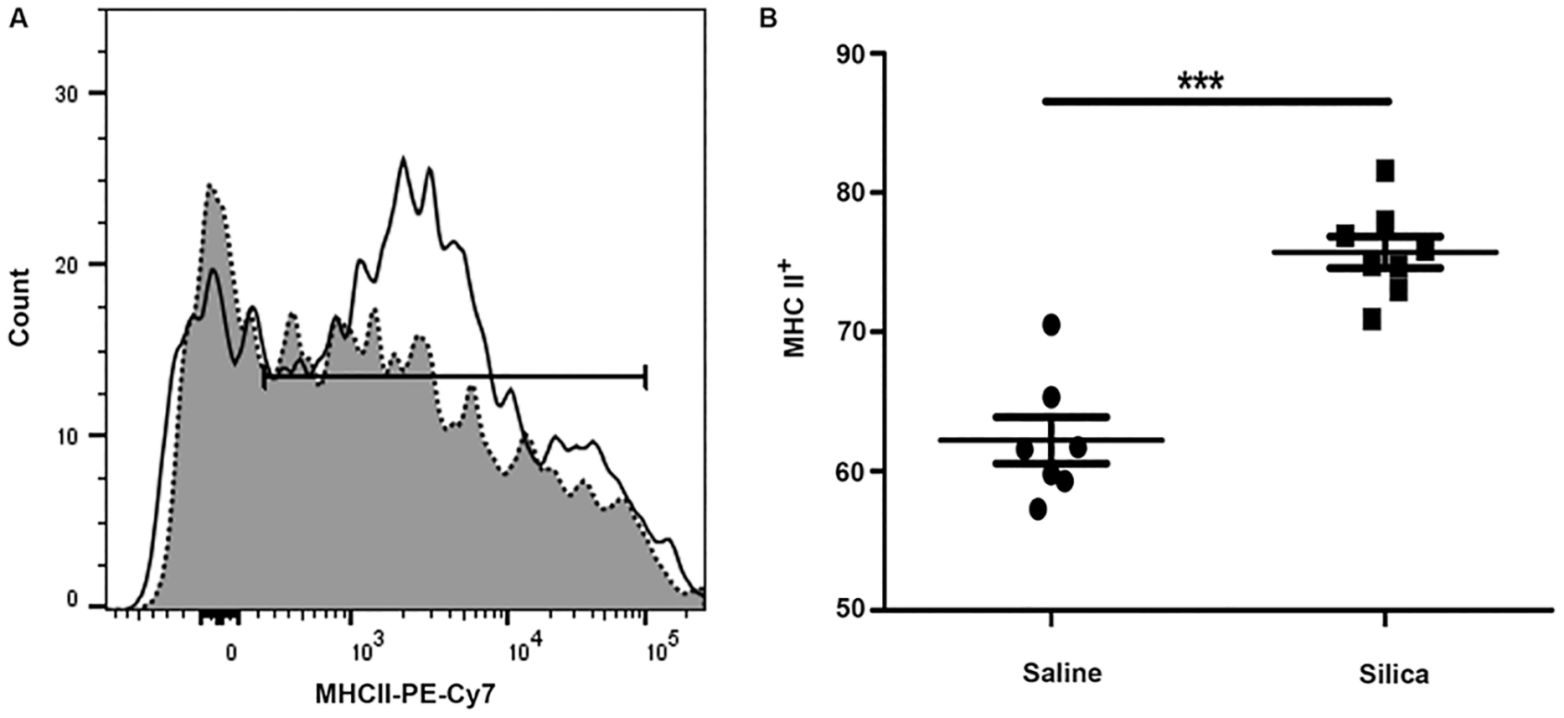
Expression of major histocompatibility complex II (MHC II) on the CD11b^+^/CD11c^+^ subset of CFPs in silica-induced pulmonary fibrosis. Flow cytometric analysis of MHC II expression. (A) Representative histogram of MHC II expression on sized, single, live, CD45^+^/DDR2^+^, CD11b^+^/CD11c^+^ cells in lung digest from saline-instilled (gray fill, dotted line) or silica-instilled animals (black line) eight-weeks after instillation. (B) Quantification of MHC II expression based on gating strategy as described above from lung digest of saline- or silica-instilled animals.

Immune populations such as dendritic cells and other antigen presenting populations exhibit increased expression of co-stimulatory molecules upon exposure. Given that the CD45^+^DDR2^+^ CFP expresses markers associated with these immune populations, co-stimulatory molecule expression was assessed on CD45^+^DDR2^+^ CFPs that expressed CD11b^+^CD11c^+^ (immune CFP subset) from lung digest of silica- or saline-instilled animals. The co-expression of CD80/CD86 by this immune CFP subset was significantly increased in the fibrotic lungs of silica-exposed animals versus saline controls (27.38% vs. 13.68%, **p = 0.0011) (Fig 3A, saline 3B, silica, quantified in 3C). Upregulation of MHC II is generally concomitant with upregulation of immune co-stimulatory molecules in response to exposures, therefore the expression of MHC II on this population was also examined. With silica-induced pulmonary fibrosis, there was increased expression of MHC II (75.71% silica vs. 62.21% saline, ***p = <0.0001) on the CD11b^+^CD11c^+^ subset of CFPs (Fig 4A, quantified in 4B).

### Functional immune role of the CD11b^+^CD11c^+^ subset of CFPs in silica-induced pulmonary fibrosis

**Fig 5:**
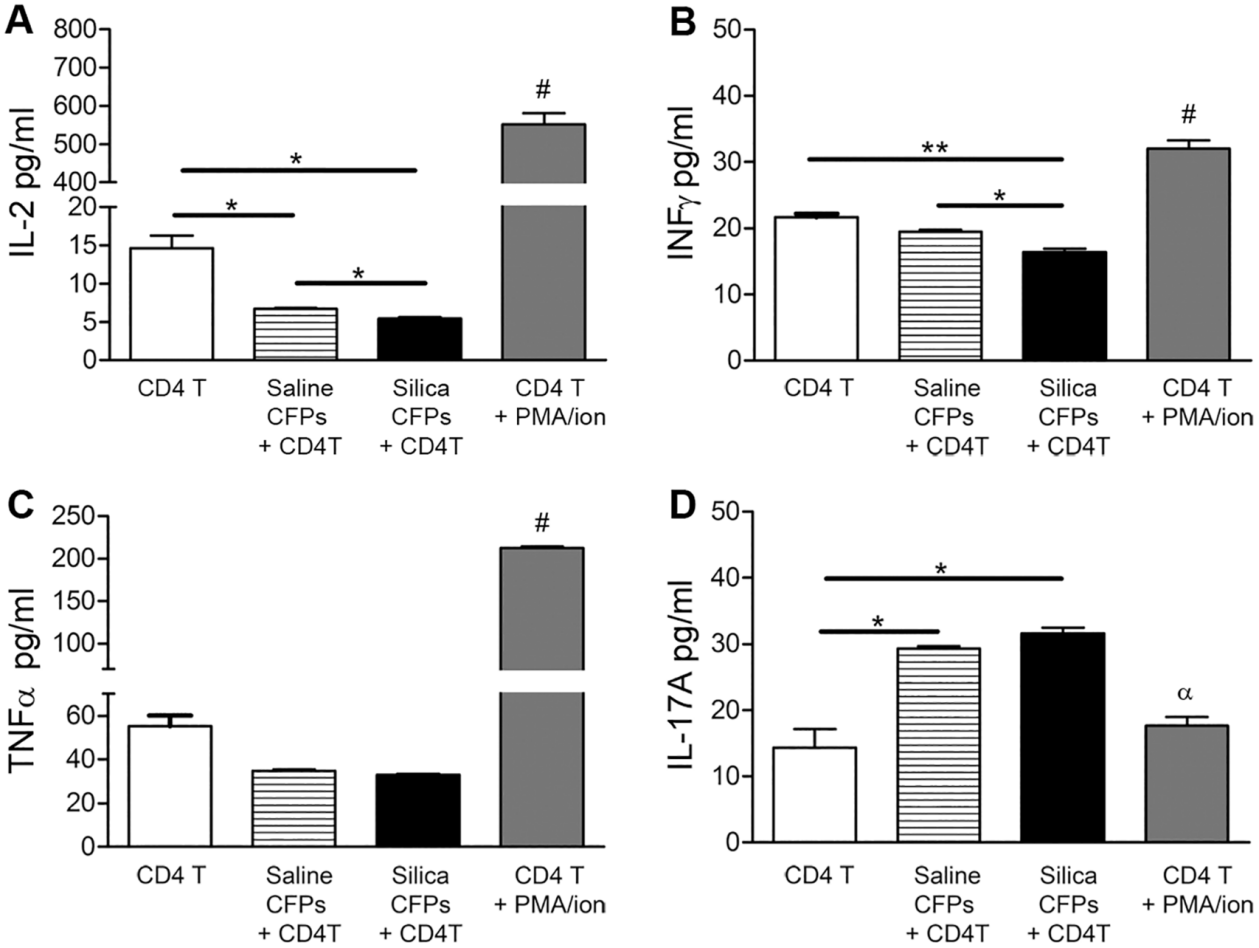
Functional role of the CD11b^+^/CD11c^+^ subset of CFPs on CD4^+^ T cell cytokine expression profiles. Flow cytometric bead array of supernatant from co-cultures of sorted CD11b^+^/CD11c^+^ CFPs from saline- or silica-instilled lung digest with isolated CD4^+^ T cells demonstrated expression of (A) Interleukin-2 (IL-2), (B) Interferon gamma (IFN-γ), (C) Tumor necrosis factor-alpha (TNF-α), (D) Interleukin-17A (IL-17A). CD4^+^ T cells alone and stimulated CD4^+^ T cells served as controls (A-D). Statistical significance versus all other bars indicated by #; α indicates significance versus co-cultures; * p≤0.05, ** p≤0.005.

**Fig 6:**
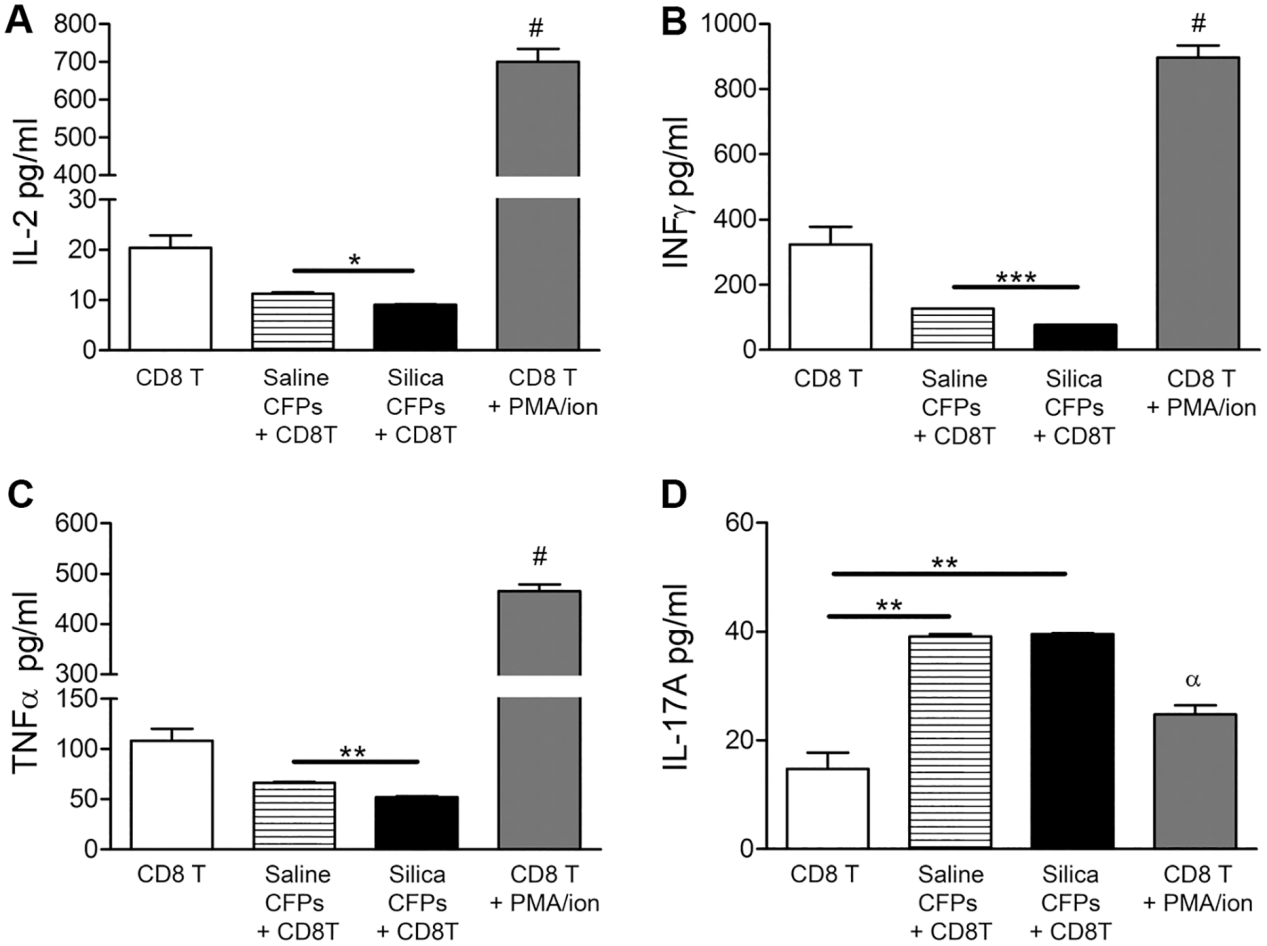
Functional role of the CD11b^+^/CD11c^+^ subset of CFPs on CD8^+^ T cell cytokine expression profiles. Flow cytometric bead array of supernatant from co-cultures of sorted CD11b^+^/CD11c^+^ CFPs from saline- or silica-instilled lung digest with isolated CD8^+^ T cells demonstrated expression of (A) Interleukin-2 (IL-2), (B) Interferon gamma (IFN-γ), (C) Tumor necrosis factor-alpha (TNF-α), (D) Interleukin-17A (IL-17A). CD8^+^ T cells alone and stimulated CD8^+^ T cells served as controls (A-D). Statistical significance versus all other bars indicated by #; α indicates significance versus co-cultures; * p≤0.05, ** p≤0.005, *** p≦0.0005.

In order to determine the functional effects of this novel CD11b^+^CD11c^+^ subset of CFPs on CD4^+^ and CD8^+^ T cell populations, CD11b^+^CD11c^+^ CFPs were sorted from the lungs of saline or silica treated animals at eight weeks post-instillation. Sorted cells were co-cultured with naïve CD4^+^ or CD8^+^ T cells isolated from untreated animals in a ratio of 1:10. Following 48 hour co-culture, supernatant was collected and analyzed by flow cytometric multiplex bead array for expression of T cell-associated cytokines (Figs 5 and 6). T cell expression of interleukin-2 (IL-2), interferon-gamma (IFN-γ), TNF-alpha (TNF-α), and interleukin-17A (IL-17A) were examined. As a control, CD11b^+^CD11c^+^ CFPs (1x10^4^ cells) were plated alone, and demonstrated no detectable expression of the cytokines examined. There was a statistically significant decrease in the production of IL-2 by CD4^+^ T cells co-cultured with CD11b^+^CD11c^+^ CFPs from silica-instilled (5.450 pg/ml) versus saline-instilled lungs (6.715 pg/ml, *p = 0.0318) (Fig 5A). Production of IL-2 by CD4^+^ T cells co-cultured with CD11b^+^CD11c^+^ CFPs from silica instilled animals was also significantly less than produced by stimulated (PMA/ionomycin treated) CD4^+^ T cells (551.9 pg/ml, ***p<0.0001) (Fig 5A). In addition, IFN-γ expression by CD4^+^ T cells was decreased in co-cultures with CD11b^+^CD11c^+^ CFPs from silica-treated animals (16.40 pg/ml) versus saline controls (19.50 pg/ml, *p = 0.0405, Fig 5B). This was also significantly less than observed with stimulated CD4^+^ T cell controls (16.40 pg/ml vs. 32.07 pg/ml, respectively, ***p = 0.0004, Fig 5B).

Examination of pro-inflammatory factors revealed that TNF-α production by CD4^+^ T cells was not significantly altered in co-cultures with CD11b^+^CD11c^+^ CFPs from silica-treated animals versus control, (32.94 pg/ml vs. 34.86 pg/ml, p = 0.1056, Fig 5C) nor was IL-17A expression significantly altered in CD4^+^ T cell co-cultures with CD11b^+^CD11c^+^ CFPs from silica versus saline animals (31.60 pg/ml vs. 29.35 pg/ml, p = 0.1406, Fig 5D). However, IL-17A production by CD4^+^ T cells was significantly increased in co-cultures with CD11b^+^CD11c^+^ CFPs regardless of fibrosis compared to the level produced by stimulated CD4^+^ T cell controls (31.60 pg/ml (silica) or 29.35 pg/ml (saline) vs. 17.70 pg/ml, **p = 0.0011 and **p = 0.0026, respectively) (Fig 5D).

Analysis of the impact of the CD11b^+^CD11c^+^ subset of CFPs on CD8^+^ T cells showed that co-culture of CD11b^+^CD11c^+^ CFPs from animals with silica-induced pulmonary fibrosis resulted in decreased IL-2 production by CD8^+^ T cells (9.130 pg/ml) versus saline-instilled control animals (11.27 pg/ml, respectively, *p = 0.0223, Fig 6A). Similarly, CD8^+^ T cells from co-cultures with CD11b^+^CD11c^+^ CFPs from silica versus saline treated animals resulted in decreased production of IFN-γ (127.5 pg/ml vs. 77.48 pg/ml, ***p = 0.0002, Fig 6B).

CD8^+^ T cells also produced significantly less TNF-α in co-cultures with the CD11b^+^CD11c^+^ CFP subset from silica mice versus saline control animals (66.59 pg/ml vs 52.15 pg/ml, **p = 0.090, Fig 6C). While CD8^+^ T cell production of IL-17A was not significantly altered in co-cultures with CD11b^+^CD11c^+^ CFPs from silica versus saline-treated animals (39.53 pg/ml vs. 39.11 pg/ml, p = 0.4655, Fig 6D), IL-17A was significantly increased in co-cultures with CD11b^+^CD11c^+^ CFPs versus stimulated CD8^+^ T cell controls (39.53 pg/ml (silica) or 39.11 pg/ml (saline) vs. 24.78 pg/ml, p = **0.0029, **p = 0.0034, respectively, Fig 6D).

## Discussion

Current therapies for the treatment of pulmonary fibrosis merely act to slow disease progression without cure, or involve risky transplantation and life-long anti-rejection treatment. While an active area of ongoing research, data is conflicting with regard to targeting the immune system for the treatment of pulmonary fibrosis. While there is known involvement of the immune response in fibrosis, it is unclear whether immune suppression or immune activation is beneficial to treatment of the disease. In the clinical setting in the treatment of the idiopathic form of pulmonary fibrosis, immunotherapies achieved little to no therapeutic response thus far [4, 13]. However, early attempts at immune based therapies were broadly targeted, administered late in disease, and dose and combination therapies complicated outcomes. Due to the known contribution of the inflammatory and immune response to exposures, an immune-based therapy that is *specifically* targeted may, in fact, prove to be effective in slowing or reversing pulmonary fibrosis. Our previous studies have led to the identification of an hematopoietic stem cell (HSC)-derived circulating fibroblast precursor (CFP) that is present in the lungs and contributes to the collagen producing pulmonary fibroblast population. The CFP population is unique in its co-expression of CD45 and the collagen receptor (DDR2)–potentially enabling these cells to both sense and respond to a changing matricellular environment. In the present study we demonstrate a role for a novel immune subset of the CFP population in silica-induced pulmonary fibrosis. Together these studies indicate a unique dual role for CFPs, contributing to immune function in lung as shown herein, in addition to contributing to a population of lung fibroblasts [10] suggesting that the CFP may be a valuable therapeutic target toward inhibition of fibrotic progression.

To examine the immune role of this novel population in pulmonary disease, we employed a silica-induced model of pulmonary fibrosis. Inhalation of silica particles results in a progressive, persistent, and robust, pro-fibrotic response in rodents [14]. This model is clinically relevant in both its non-resolving nature, and in the development of fibrotic nodules which resemble those observed in patients with exposure to occupational dusts and particulates [14]. Studies have shown that exposure to silica nanoparticles *in vitro* results in alterations in surface marker expression of CD54, CD80, CD86 and MHC II by immune cell populations [15]. Therefore, a silica-induced model of pulmonary fibrosis was employed in order to provide the first direct flow cytometric and functional evaluation of the immunological role of the novel CD11b^+^CD11c^+^ CFP subset. Based on our findings, the CFP population is skewed toward the CD11b^+^CD11c^+^ immune subset in the fibrotic disease state. This subset of CFPs exhibits a CD11b^high^ phenotype which is suggestive of a monocytic/immature dendritic-like population, or a precursor closely related to a monocyte [16]. In addition, this population exhibits a CD11c^low^ profile in disease, which may also indicate that an immature state of these cells is maintained in the lung even in advanced fibrosis (8 weeks). Further, an increase in the immune subset of CFPs expressing MHC II and co-stimulatory molecules CD80 and CD86 was observed, indicating that these CFPs exhibit an activated immune phenotype in exposure-induced fibrosis.

Several other groups have examined immune populations during development of pulmonary fibrosis in murine models. A study by Holian et. al. using a silica-induced model of pulmonary fibrosis examined the dynamics of antigen presenting populations in the lung during fibrotic progression [17]. This study found that there were early increases in the CD11c^hi^/MHC II^hi^ population of dendritic cells and early declines in the CD11c^hi^/MHC II^lo^ alveolar macrophage population. However, by 28 days post silica-instillation these populations had returned to levels consistent with controls and their studies did not reveal any changes in CD86 co-stimulatory molecule expression on either population following silica exposure. However, studies in a bleomycin model of pulmonary fibrosis by Soler’s group demonstrated a sustained increase in CD11c^+^/MHC II^+^ dendritic cells and increased expression of MHC II and CD86 throughout the fibrotic stage of bleomycin-induced disease [18]. Interestingly, the novel immune CFP subset identified herein was elevated at 8 weeks post silica-instillation and demonstrated significant increases in MHC II and co-stimulatory molecule expression, indicating either a unique immune signature of the CFP in response to silica-induced fibrosis or a cyclical immune reaction that may perpetuate the fibrotic response.

While the mechanism(s) driving the activated phenotype of the immune CFP subset are not yet known, studies by Lee et. al. have demonstrated that immature bone marrow-derived dendritic cells upregulate expression of CD86 in response to collagen exposure through DDR2 signaling [5]. Our studies have shown increased deposition of collagen with silica-induced pulmonary fibrosis, which is concurrent with the increased percentage of the CD11b^+^CD11c^+^ subset of CFPs expressing CD80/CD86, suggesting that DDR2/collagen I signaling may play a role in activation of the immune CFP subset.

In addition to phenotypic changes in response to silica-induced pulmonary fibrosis, herein we demonstrate that the immune CFP subset functionally contributes to the immune microenvironment by promoting T cell skewing. Data presented demonstrate that pulmonary CFPs from silica-induced pulmonary fibrosis exhibit an inhibitory role toward the Th1 phenotype based on decreased production of IL-2 and IFN-γ by CD4^+^ and CD8^+^ T cells. Data also demonstrate promotion of a pro-inflammatory T cell phenotype based on production of TNF-α and IL-17A. This T cell cytokine profile has been associated with fibrotic progression (reviewed in [19]). Notably, the CD11b^+^CD11c^+^ CFP subset promoted the production of IL-17A by both CD4^+^ and CD8^+^ T cells in both control and silica-instilled animals, suggesting that CFPs may skew toward an inflammatory Th17 phenotype based on production of this Th17-associated cytokine. IL-17 production by T cells has been implicated in the pathogenesis of pulmonary fibrosis [20, 21], (reviewed in [4]). IL-17 was also shown to be elevated in the bronchoalveolar lavage of patients with idiopathic pulmonary fibrosis [17] and in a bleomycin animal model of pulmonary fibrosis [22], reviewed in [4]. Together, data demonstrate that the CFP subset skews the lung immune environment away from a Th1 phenotype and toward a pro-inflammatory environment in silica-induced pulmonary fibrosis which may contribute to fibrotic development and progression.

It has been demonstrated that bone marrow-derived populations are largely recruited in diseases exhibiting rapid turnover in the lung, especially in cases of chronic injury (reviewed in [23]). This suggests that the HSC-derived CFP population may play a significant role in the development and progression of pulmonary fibrosis. Importantly, the immune subset of this population has not previously been described, nor have the contribution(s) of this novel DDR2-expressing population to pulmonary fibrosis ever before been investigated. The discovery that a subset of CFPs is capable of immune modulating effects in disease is important in terms of our understanding of the plasticity of such populations depending on micro-environmental stimuli. Future examination of the mechanisms by which the CFP senses and responds to the matricellular and microenvironmental stimuli of the fibrotic lung may provide insight into the pathways driving the dual roles of the CFP and may uncover a pathobiological mechanism critical to the fibrotic response.
